# Odor Concentration Change Coding in the Olfactory Bulb

**DOI:** 10.1523/ENEURO.0396-18.2019

**Published:** 2019-02-27

**Authors:** Ana Parabucki, Alexander Bizer, Genela Morris, Antonio E. Munoz, Avinash D. S. Bala, Matthew Smear, Roman Shusterman

**Affiliations:** 1 Sagol Department of Neurobiology, University of Haifa, Haifa 3498838, Israel; 2 Institute of Neuroscience, University of Oregon, Eugene, OR 97403; 3 Department of Psychology, University of Oregon, Eugene, OR 97403

**Keywords:** contrast, dynamical stimulus, electrophysiology, mitral and tufted cells, olfactory bulb

## Abstract

Dynamical changes in the environment strongly impact our perception. Likewise, sensory systems preferentially represent stimulus changes, enhancing temporal contrast. In olfaction, odor concentration changes across consecutive inhalations (*ΔC_t_*) can guide odor source localization, yet the neural representation of *ΔC_t_* has not been studied in vertebrates. We have found that, in the mouse olfactory bulb, a subset of mitral/tufted (M/T) cells represents *ΔC_t_*, enhancing the contrast between different concentrations. These concentration change responses are direction selective: they respond either to increments or decrements of concentration, reminiscent of ON and OFF selectivity in the retina. This contrast enhancement scales with the magnitude, but not the duration of the concentration step. Further, *ΔC_t_* can be read out from the total spike count per sniff, unlike odor identity and intensity, which are represented by fast temporal spike patterns. Our results demonstrate that a subset of M/T cells represents *ΔC_t_*, providing a signal that may instruct navigational decisions in downstream olfactory circuits.

## Significance Statement

As an animal tracks an odor plume, concentration changes over time. Here, we show that olfactory bulb neurons explicitly represent concentration changes between consecutive inhalations. This response property enhances temporal contrast, as in other sensory systems. Fine temporal spike patterns do not improve concentration change decoding. These signals may guide olfactory navigation in the natural environment.

## Introduction

The brain must track how external information changes with time (Wertheimer, 1912; Bregman, 1994). Correspondingly, sensory circuits deploy specialized cell types for dynamic stimuli: visual neurons emphasize luminance changes and motion (Ratliff et al., 1963), auditory neurons capture amplitude and frequency modulation (Langner, 1992), and somatosensory neurons encode vibrating touch (Werner and Mountcastle, 1965; Mountcastle et al., 1967). For many animals, odor concentration changes are equally relevant, since they carry information about odor source location (Murlis et al., 1992; Ache et al., 2016). Vertebrates can localize odor sources either by comparing between simultaneous samples from the two nostrils, or by comparing samples taken sequentially from different locations (Catania, 2013). When bilateral sampling is prevented by naris occlusion, animals are only partly impaired at localizing odor sources (Porter et al., 2007; Khan et al., 2012; Catania, 2013; Jones and Urban, 2018). Therefore, vertebrates must also sense changes of odor concentration, from sniff to sniff (*ΔC_t_*), to guide them to an odor source. Yet despite this evidence that *ΔC_t_* can guide odor tracking, whether olfactory neurons encode sniff to sniff changes has not been directly addressed.

Unlike invertebrate olfactory systems, in which olfactory sensory neurons (OSNs) are continuously exposed to the medium, air-breathing vertebrates discretize the input to OSNs into intermittent inhalations. In this case, the brain must maintain a memory of odor concentration across the exhalation interval to compute *ΔC_t_*.

How and where does the olfactory system solve this problem? We demonstrate here that a subset of neurons in the olfactory bulb encode *ΔC_t_* on the time scale of a single sniff. Thus, like their counterparts in other sensory systems such as ON/OFF responses in vision (Kuffler, 1953; Schiller, 1992; Westheimer, 2007), a subset of olfactory neurons represents stimulus increments and decrements. Further, these representations depend on the magnitude of the concentration step, but not the duration of the step (i.e., for how many sniffs it lasts). Lastly, while fast temporal spike patterns can improve decoding of absolute concentration, concentration changes can be read out from total spike count.

## Materials and Methods

### Animals

Data were collected in seven C57BL/6J male mice. Subjects were 8–16 weeks old at the beginning of recordings and were maintained on a 12/12 h light/dark cycle (lights on at 8 P.M.) in isolated cages in an animal facility. All animal care and experimental procedures were in accordance with a protocol approved by the University of Haifa and University of Oregon Institutional Animal Care and Use Committees.

### Surgery

Mice were anesthetized using isofluorane gas anesthesia, and a head plate and a pressure cannula were implanted. For sniffing cannula implantation, we drilled a small hole in the nasal bone, into which the thin 7- to 8-mm-long stainless-steel cannula (gauge 23 capillary tubing, Small Parts) was inserted, fastened with glue, and stabilized with dental cement (Verhagen et al., 2007). A small craniotomy was performed above one of the olfactory bulbs, contralateral to the side of sniffing cannula implantation. The reference electrode was implanted in cerebellum. At the end of the procedure, the craniotomy was covered with a biocompatible silicone elastomer sealant (Kwik-cast, WPI). The mice were given 3 d after a surgery for recovery.

### Odor delivery

**Figure 1.**
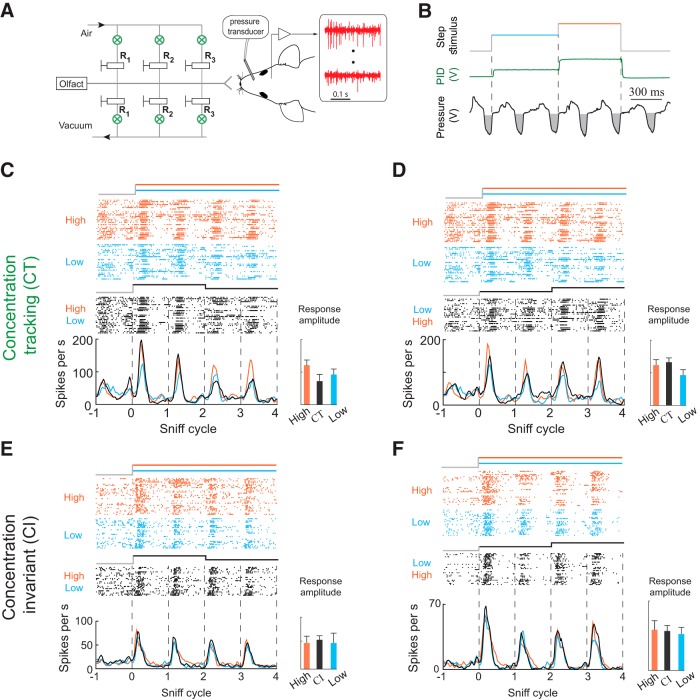
*CT* and *CI* odor responses. ***A***, Schematic of the experiment. Right, A head-fixed mouse implanted with an intranasal cannula and a multi-electrode chamber was positioned in front of the odor delivery port. Left, CCM. ***B***, Odor concentration step paradigm. Odor concentration changes every two sniff cycles. Green curve indicates the response of a photoionization detector (PID) to presentation of ethyl acetate. Sniff waveforms (black) are shown below the plots. Gray areas indicate inhalation. Vertical dashed lines indicate onset of concentration changes. ***C***, ***D***, Examples of *CT* responses from two cells. Raster and PSTH plots of M/T cell response to static high concentration (orange), static low concentration (blue), and concentration step stimuli (black). The responses of these cell odor pairs change with odor concentrations the same way in both static and step stimuli. Bar graph on right shows peak response amplitudes on the third sniff cycle for each stimulus. Error bars indicate SD (see Materials and Methods). ***E***, ***F***, Same as ***C***, ***D*** but for two cell-odor pairs that are invariant to odor concentration in the presented range.

For stimulus delivery, we used a custom eight-odor air dilution olfactometer, based on a previous design (Bodyak and Slotnick, 1999). When no odor was being presented to the mouse, a steady stream of clean air (1000 ml/min) was flowed to the odor port. During odorant presentation, N_2_ flowed through the selected odorant vial. We used multiple odorants obtained from Sigma-Aldrich. The odorants were stored in liquid phase (diluted either 1:5 or 1:10 in mineral oil) in dark vials. We used acetophenone, amyl acetate, geraniol, ethyl acetate, S-limonene, methyl butyrate, menthone, methyl salicylate, pentyl acetate, and vanillin as odorants. The odorant concentration delivered to the animal was reduced additional tenfold by air dilution and homogenized in a long Teflon tube before reaching the final valve. After sufficient mixing and equilibration time, the dual three-way Teflon valve (SH360T042; NResearch) directed the odor flow to the odor port and diverted the clean airflow to the exhaust. All air flows and line impedances were equalized to minimize the pressure transients resulting from odor and final valve switching. The time course of odor concentration was checked by Photo-Ionization Detector (200B mini-PID; Aurora Scientific). The concentration reached a steady state ∼40 ms after final valve opening (Resulaj and Rinberg, 2015). Further, to change odor concentration, we passed stable odorized airflow through a concentration change manifold (CCM; Fig. 1*A*). Odor concentration changes were achieved by activating a pair of matching solenoids (LHQA2411220H; The Lee Company) which performed air dilution. For each pair of solenoids, one valve was connected to a vacuum channel and the other to a clean airflow channel. Solenoid activation in the vacuum channel diverted part of the odorized air, while solenoid activation in the air channel contributed an equal amount of flow back into the system. To maintain constant total airflow (Extended Data Fig. 1-1*B*), the impedance of each air channel was matched to the impedance of the corresponding vacuum channel using manual needle valves R1-R3 (NV3H-1012-3-S; Beswick Engineering). To ensure that the temporal profile of odor concentration stabilized before inhalation began, we predominantly used odorants with higher vapor pressure (Martelli et al., 2013). For these high vapor pressure odorants, the stimulus reaches 95% of final concentration in 20–40 ms (Fig. 1*C*).

10.1523/ENEURO.0396-18.2019.f1-1 Figure 1-1 Download Figure 1-1, PDF file.

### Electrophysiological recording

We recorded mitral/tufted (M/T) cell activity using acute 16- or 128-channel matrix array of Si-probes (a2x2-tet-3mm-150-150-121-A16, M4x8-5mm-Buz-200/300um; NeuroNexus). Cells were recorded in both ventral and dorsal mitral cell layers. The data were acquired using a 128-channel data acquisition system (RHD2000; Intan Technologies) at 20-kHz sampling frequency. To monitor sniffing, the intranasal cannula was connected to a pressure sensor with polyethylene tubing (801000; A-M Systems). The pressure was measured using a pressure sensor (24PCEFJ6G; Honeywell). The amplified output signal from the pressure sensor was recorded in parallel with electrophysiological data on one of the analog input channels.

Before recording began, the mice were first adapted to head fixation. Mice typically remained quiescent after one to two sessions of head fixation, after which recording sessions started. We presented two to three odors in a single session in pseudo-random sequence with an average interstimulus interval of 7 s. Each odor was presented in four temporal patterns: (1) static high, high concentration (∼1–2% of saturated vapor pressure) of odor for four sniff cycles; (2) static low, low concentration (50% of high concentration level) for four sniff cycles; (3) a step from high to low, for the first two sniff cycles, concentration level was equal to the level of static high, after which the concentration stepped to the low concentration; and (4) a step from low to high, two sniff cycles of low concentration followed by two sniffs of high concentration. We controlled odor concentration using a CCM. Odor onsets and concentration changes were triggered at the beginning of the exhalation phase, which occur at positive-going zero crossings of the pressure signal. Since odor cannot orthonasally enter the nose during exhalation, triggering by exhalation onset allows enough time for the odor stimulus to reach a steady state of concentration by the time the animal begins to inhale. One session usually lasted for 60–90 min and consisted of 300–400 trials.

### Spike extraction and data analysis

All analysis was done in MATLAB (MathWorks). Electrophysiological data were filtered between 300 Hz and 5 kHz and spike sorted. For spike sorting we used software package written by Alex Koulakov (Shusterman et al., 2011; Table 1)

**Table 1.** Statistical table

	Data structure	Type of test	Power
If cell is responsive to an odor	Fitted data, non-normal	Kolmogorov–Smirnov test	*p* < 0.005
ROC analysis CI	Fitted data, normal	*t* test	*p* = 0.63
ROC analysis CT	Fitted data, normal	*t* test	*p* = 0.08
ROC analysis +/*–ΔC_t_*	Fitted data, normal	*t* test	*p* < 0.001
Spike count contrast enhancement for +/*–ΔC_t_*	Fitted data, non-normal	Wilcoxon signed-rank test	*p* = 7.57e-5 for *+ΔC_t_* responses*p* = 8.64e-5 for *–ΔC_t_* responses
Spike count contrast enhancement for CT	Fitted data, non-normal	Wilcoxon signed-rank test	*p* = 0.20
Spike count contrast enhancement for CI	Fitted data, non-normal	Wilcoxon signed-rank test	*p* = 0.18
Peak amplitude contrast enhancement for +/*–ΔC_t_*	Fitted data, non-normal	Wilcoxon signed-rank test	*p* = 2.41e-6 for *+ΔC_t_* responses*p* = 2.08e-8 for *–ΔC_t_* responses
Peak amplitude contrast enhancement for CT	Fitted data, non-normal	Wilcoxon signed-rank test	*p* = 0.97
Peak amplitude contrast enhancement for CI	Fitted data, non-normal	Wilcoxon signed-rank test	*p* = 0.21
*ΔC_t_* sensitivity is step magnitude dependent	Normal distribution	*t* test	1.25-fold change: *p* = 0.72;1.5-fold change: *p* < 0.01;2-fold change: *p* < 0.01
*ΔC_t_* sensitivity is step duration independent	Normal distribution	Wilcoxon test	For spike count *p* = 0.08;for peak amplitude *p* = 0.12

### Temporal alignment of responses

For analysis, sniffing traces were down-sampled to 1 kHz, and filtered in the range of 0.5–30 Hz. The inhalation onset and offset were detected by zero crossings of a parabola fit to the minima of the pressure signal following the onset of the inhalation. Inhalation onset/offset was defined as the first zero crossing of the parabola (Shusterman et al., 2011). We defined two intervals: the first is from inhalation onset to inhalation offset and the second is the rest of the sniffing cycle, from the inhalation offset to the next inhalation onset. While the duration of the first interval is concentration independent, the duration of the second interval depends on the concentration of presented odor (Extended Data Fig. 5-1). To compare neuronal responses across trials and concentrations, we morphed the inhalation part of the sniff cycle and corresponding spike train to the average one (Shusterman et al., 2011). The second part of the sniff cycle and corresponding neural activity were matched to the average over trials: longer cycles were truncated and shorter were zero padded.

### Odor responses

To establish whether a cell is responsive to an odor, we compared the cumulative distribution of the neuronal spikes without odors to the cumulative distribution of neuronal activity during the first odorized sniff cycle, using the Kolmogorov–Smirnov test. Neuronal activity without odor was sampled from three sniffs preceding odor delivery across all trials. Neuronal activity for a given odor was sampled from the first sniff after stimulus onset. Cells were considered responsive if the distribution of spiking activity during the first odorized cycle statistically differed from the distribution of baseline responses in at least one 10-ms bin relative to inhalation onset (*p* < 0.005; Benjamini–Hochberg multiple comparison correction) or if their average spike rate over the sniff cycle differed significantly from baseline (*p* < 0.05).

### Recovery index (RI)

To measure how Δ*C_t_* cell-odor pairs recover in sniffs after the concentration step, we quantified a RI, using the peak amplitude of the response. For positive *ΔC_t_* responses, it consists of the ratio between change of response between two consecutive sniff cycles after the concentration change (LH3-LH4) to the difference between *ΔC_t_* response and the response on the matching static stimulus (LH3-H3):

$$\frac{LH3-LH4}{LH3-H3}$$

If the M/T cell responds with identical amplitude on two sniff cycles following the concentration step, this will lead to LH4 = LH3, the numerator will be zero and thus RI = 0. In the other limiting case, when the response on the second sniff following the step (LH4) is equal to the static response (H3), the denominator will be equal to the numerator and therefore RI will be equal 1. Therefore, most of the RIs will be distributed between 0 and 1.

By analogy, for negative Δ*C_t_* responses, RI will take the following form:

$$\frac{HL3-HL4}{HL3-L3}$$

### ROC analysis of contrast enhancement

ROC analysis provides a measure of how well a given cell-odor pair can discriminate between two stimuli. To measure the discriminability between the static odor stimuli, high and low, we compute the area under the ROC curve (auROC) for the distributions of spike counts over the third sniff of each stimulus (Extended Data Fig. 4-2*A1*,*B1*,*C1*; Green and Swets, 1966). ROC curves were created by plotting the probability that the single-trial spike count (Extended Data Fig. 4-2*A2*) exceeds a given value (Extended Data Fig. 4-2*A3*,*B3*,*C3*) for two stimulus types. For each point, on the *x*-axis is the probability for one stimulus type, on the *y*-axis is the probability for another stimulus type. Dark curves show the probabilities for dynamic versus static, while lighter curves show the probabilities of static high versus static low. An auROC value of 1 indicates no overlap between the two distributions, while a value of 0.5 indicates complete overlap between the two distributions. We then plot the static stimulus auROC against the *ΔC_t_* discriminability (Extended Data Fig. 4-2*D*,*E*). This plot shows whether a given cell-odor pair shows contrast enhancement between concentrations during step stimuli.

Three example cell-odor pairs are shown in such a plot (Extended Data Fig. 4-2*A–C*). *CI* responses do not discriminate between high and low concentration (*t* test, *p* = 0.63), and give values of near 0.5 for both static and flickering stimuli (Extended Data Fig. 4-2*E*). CT responses discriminate equally well between static and flickering stimuli, and thus fall along the diagonal of this plot (*t* test, *p* = 0.08). *ΔC_t_* responses discriminate better between dynamic and static stimuli than between two static stimuli, so that they fall above the diagonal (*t* test, *p* < 0.001 for both *+ΔC_t_* and *–ΔC_t_*). These analyses demonstrate that *ΔC_t_* sensitivity enhances the contrast between concentrations, potentially facilitating detection of concentration change.

### Odor concentration classification analysis

To estimate how well single neurons (*n* = 49) can discriminate between two odor concentrations on a trial by trial basis, we constructed a Mahalanobis distance linear classifier. For concentration discrimination, we calculated discriminability between responses to static high and static low on the 3rd sniff cycle, L_3_ and H_3_. For every cell and for every pair of concentrations we counted spikes using multiple time bins (5, 10, 20, 40, 80, and 160 ms). Single trials were randomly selected and compared to a set of templates constructed from 70% of trials for each of the two concentrations. We used the *mahal* function in MATLAB to estimate Mahalanobis distance from each single trial vector to two groups of multiple trial templates representing two concentrations. This procedure was repeated 300 times for different single trial population vectors and was repeated for each bin size.

A similar analysis was performed on the same cell-odor pairs to estimate discriminability in *ΔC_t_*. For *ΔC_t_* discrimination, we calculated discriminability between LH_3_ and L_3_ sniffs for *+ΔC_t_* responses and HL_3_ and H_3_ sniffs for *–ΔC_t_* responses.

### Behavioral experiments

Two mice were implanted with a head bar and a cannula in their nose, both secured to the skull by dental cement (Smear et al., 2011). After recovery from surgery, mice were water restricted so that they are motivated to work for water reward during behavioral testing.

To measure *ΔC_t_* sensitivity, we used a Go/No-Go paradigm (Smear et al., 2011). Trial events were controlled and behavioral outputs (sniffing and licking) were measured using MATLAB and a custom-built Arduino-based behavior-control system. Stimulus presentation is synchronized to the sniff cycle, such that changes in odor concentration only occur while the animal is exhaling. Thus, there is no change in odor concentration during inhalation, and the animal must compare two discrete odor samples across time to detect any changes in odor concentration.

Mice were initially trained in a simple odor detection task, in which they are supposed to lick when odor is presented, and not lick when a blank stimulus occurs. After they have acquired at least 90% performance in this task, they begin *ΔC_t_* training. In the second phase of training, mice were trained to report positive or negative *ΔC_t_* relative to an absolute concentration, *C*. All trials start by delivering the baseline concentration *C* to the subject during the first sniff. During the second sniff, however, the concentration can either change (*C+ΔC_t_* or *C–ΔC_t_*; No-Go trials) or stay at *C* (Go trials). Trials containing the *ΔC_t_* signal are used as No-Go trials, because in a Go/No-Go task most errors are false alarms. By delivering *ΔC_t_* stimuli during No-Go trials, we ensure that the majority of errors occur during *ΔC_t_* trials, making these trials more informative. Responses are classified into correct-hits (H: Go trial, mouse licks), correct rejections (CR: No-Go trial, mouse does not lick), and incorrect-false alarms (FA: No-Go trial, mouse licks) and misses (M: Go trial, mouse does not lick).

## Results

### Experimental setup and response types

We recorded respiration and M/T cell activity (seven mice, 92 cells, 242 cell-odor pairs) in awake, head-fixed mice (Fig. 1*A*). To rapidly change odor concentration, we developed a novel CCM, with which rapid concentration changes were achieved by air dilution (Fig. 1*A*; Materials and Methods). Sniffing was measured through an intranasal pressure cannula (Fig. 1*A*). Using real-time closed-loop odor presentation, we switched odor concentrations at the beginning of the exhalation phase so that the stimulus reached its new steady state concentration before the onset of the next inhalation (Fig. 1*B*; Extended Data Fig. 1-1).

In most experiments, we presented odorants in two static concentration patterns: high (H) and low (L), and two dynamic patterns: a step from high to low (HL) and a step from low to high (LH). The high concentration was twice that of the low concentration, a concentration difference that is within the range of concentration changes that would be encountered in turbulent plumes (Crimaldi et al., 2002; Gaudry et al., 2012; Gire et al., 2016). Behavioral testing in a Go/No go paradigm confirmed that 2-fold concentration steps are perceptible to mice (Extended Data Fig. 1-2).

**Figure 2.**
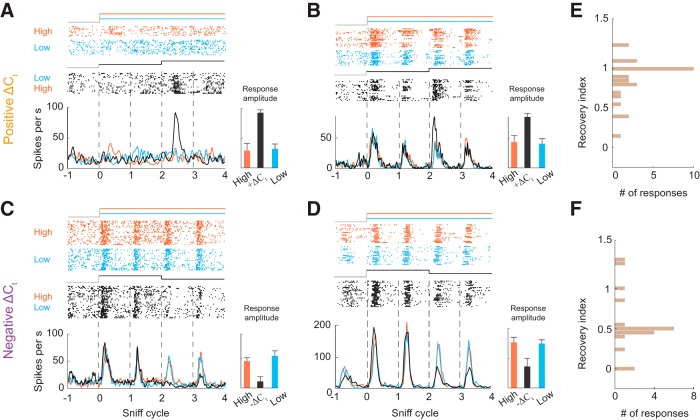
M/T cells responsive to changes in odor concentration. ***A***, ***B***, Examples of +*ΔC_t_* responses. Raster and PSTH plots of two M/T cell’s responses to static high concentration (orange), static low concentration (blue), and low to high (black). Bar graph on right shows peak response amplitudes on the third sniff cycle for each stimulus. Error bars indicate SD. ***C***, ***D***, Examples of *–ΔC_t_* responses. Raster and PSTH plots of two M/T cell’s responses to static high concentration (orange), static low concentration (blue), and high to low stimulus (black). ***E***, ***F***, Distribution of recovery indices for +*ΔC_t_* and *–ΔC_t_* responses, respectively. A value of 1 indicates complete recovery to the static odor stimulus response, while a value of 0 indicates no recovery.

10.1523/ENEURO.0396-18.2019.f1-2 Figure 1-2 Download Figure 1-2, PDF file.

Step stimuli consisted of a presentation of one concentration for two sniff cycles, followed by a switch to the other concentration. These stimuli evoked three different response types across odor-cell pairs. For some cell-odor pairs, spiking responses were proportional to odor concentration on the current sniff but were not affected by odor concentration on previous sniffs. Thus, these concentration-tracking cell-odor pairs (*CT*; Fig. 1*C*,*D*) faithfully represented the concentration on each sniff. For other cell-odor pairs, the response did not change across concentrations for static or step stimuli. We refer to these as concentration-invariant (*CI*; Fig. 1*E*,*F*). These unchanging responses may be specialized for odor identification, for which concentration invariance is an important property (Wilson and Mainen, 2006; Shusterman et al., 2018; Wilson et al., 2017; Bolding and Franks, 2018). However, testing with a wider range of concentrations would be needed to fully determine these cells’ concentration response function for a given odor. Lastly, we observed responses that were sensitive to changes in odor concentration (*ΔC_t_*; Fig. 2). For these cell-odor pairs, responses to step stimuli could not be predicted from responses to static stimuli. These *ΔC_t_* cell-odor pairs responded to LH (*+ΔC_t_*responses; Fig. 2*A*,*B*; Extended Data Fig. 2-1*A*) or to HL stimuli (*–ΔC_t_* responses; Fig. 2*C*,*D*; Extended Data Fig. 2-1*A*). For example, such a cell-odor pair may exhibit an identical response to static high and static low stimuli but respond differently when these same concentrations are alternated in the HL stimuli (Fig. 2*C*,*D*). Because of this history dependence, such a response carries information about concentration change rather than the concentration per se. The majority of *ΔC_t_* responses were selective for the direction of change (41/49; Extended Data Fig. 2-2). Further, almost all *ΔC_t_* responses increased firing rate with positive concentration changes and decreased firing rate with negative changes (46/49). Strikingly, 25% of the *+ΔC_t_* responses did not respond to the initial stimulus onset (first sniff), a change from no odor to odor, but only after the upward step in concentration (7/28; Fig. 2*A*).

10.1523/ENEURO.0396-18.2019.f2-1 Figure 2-1 Download Figure 2-1, PDF file.

10.1523/ENEURO.0396-18.2019.f2-2 Figure 2-2 Download Figure 2-2, PDF file.

For a cell to reliably report *ΔC_t_* with single sniff temporal resolution, its response should only be detectably different in the sniff that immediately follows the concentration change. On the next sniff, the response should return to the level evoked by static stimuli. To quantify the extent of recovery to the static level on the second sniff after the concentration change, we devised a RI (see Materials and Methods; Extended Data Fig. 2-3). This index ranges from 1 for complete recovery to 0 for no recovery to the static stimulus response (Fig. 2*E*,*F*). While *+ΔC_t_* responses mostly recovered near to the static level (Fig. 2*E*), *–ΔC_t_* responses do not recover completely (Fig. 2*F*).

**Figure 3.**
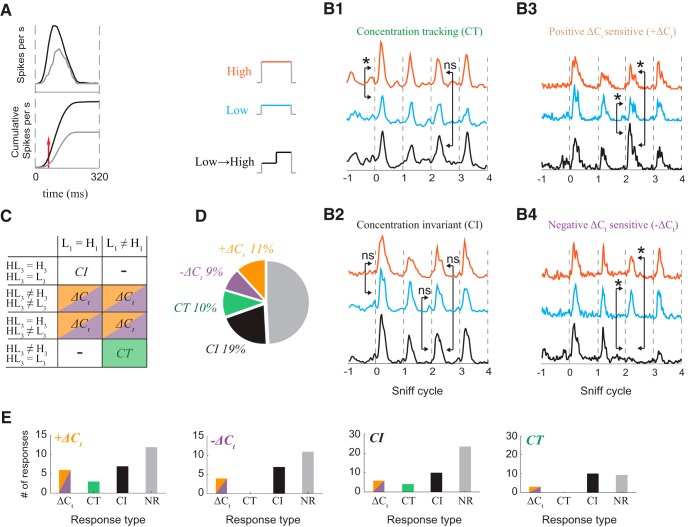
Categorization of response types. ***A***, Criteria for determining whether a cell was responsive to a given odor. Top, example of excitatory odor response PSTH. The black line is a PSTH of spiking during odorized sniffs. The gray line is a PSTH during unodorized sniffs. Bottom, cumulative spike counts of data from top plot. The red line indicates the first moment when cumulative distributions with and without stimulus become statistically different. ***B1–B4***, PSTHs from examples of each response type to high, low, and low->high stimuli are vertically separated. Arrows indicate which sniffs of the response are statistically compared. Non-significant differences are marked ns, and significant differences are marked with * (Kolmogorov–Smirnov test, *p* < 0.01). ***B1***, A *CT* cell-odor pair responded differently to the two concentrations, and this difference is not affected by a concentration step. Example data are the same as Figure 1*D*. ***B2***, A *CI* cell-odor pair responded identically to both concentrations, with or without a step. Example data are the same as Figure 1*F*. ***B3***, *ΔC_t_* cell-odor pairs responded differently to a given concentration after a concentration step. Example data are the same as Figure 2*B*. ***B4***, Example *–ΔC_t_* response data are the same as Figure 2*C*. ***C***, Comparisons used to categorize odor-cell pairs. ***D***, Distribution of different response types: *CI* (*n* = 49), *CT* (*n* = 25), positive *ΔC_t_* (*+ΔC_t_*, *n* = 28), and negative *ΔC_t_* (*–ΔC_t_*; *n* = 21). ***E***, Distribution of responses to a second odor for positive *ΔC_t_* (orange), negative *ΔC_t_* (purple), *CI* (black), and *CT* (green) cell-odor pairs.

10.1523/ENEURO.0396-18.2019.f2-3 Figure 2-3 Download Figure 2-3, PDF file.

All responses were classified as *ΔC_t_*, *CT*, or *CI*. To categorize each response, we tested whether the cumulative distribution of spike count after inhalation onset differed between stimuli (Kolmogorov–Smirnov test; Fig. 3*A*; see Materials and Methods). This statistical test is sensitive not only to changes in the total number of spikes within a sniff cycle but also to temporal redistribution of spikes within the cycle. Importantly, due to adaptation, both representation of odor concentration (Cang and Isaacson, 2003; Sirotin et al., 2015) and perception of odor intensity (Wojcik and Sirotin, 2014) depend on the duration of odor exposure. Therefore, for all analyses, we compare responses to different stimuli on the same sniff cycle after stimulus onset (e.g., we compare the 3rd sniff of the step stimulus to the 3rd sniff of the static stimulus; see Extended Data Fig. 2-4 for an example response with strong adaptation).

**Figure 4.**
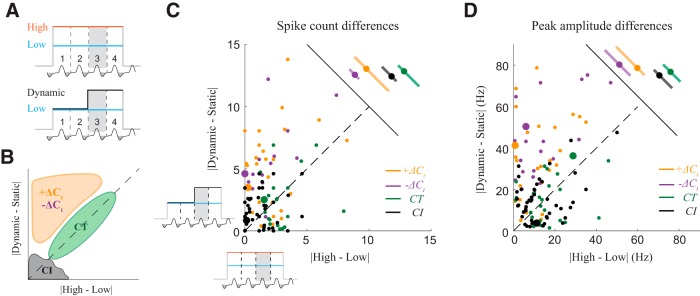
Contrast between concentrations depends on the stimulus history. ***A***, Schematic of contrast comparison. To compare contrasts, for each cell-odor pair, we take the difference in response between the 3rd sniffs of the static high (H) and static low (L) stimuli, and plot that against the difference between the 3rd sniffs of the dynamic stimulus and the corresponding static stimulus (in this example L). Thus, only the concentration in the preceding sniff varies, and the concentrations being compared are constant. ***B***, Expected distribution of responses. *CT* responses will be distributed along diagonal, *CI* responses will be distributed near the origin, and *ΔC_t_* responses will be distributed above diagonal. ***C***, Scatter plot of full sniff spike count differences between two static stimuli against differences between dynamic and static stimuli, on the 3rd sniff cycle. *CI*, *CT*, *+ΔC_t_* and *–ΔC_t_* are marked by black, green, orange, and blue color, respectively. Example cells from Figure 3*B1–B4* are indicated by enlarged dots. Adjacent panel shows the means and STDs of the spike count differences. ***D***, Same as ***C*** for differences in the peak amplitude of the response. Example cells from Figure 3*B1–B4* are indicated by enlarged dots. Adjacent panel shows the means and STDs of the peak amplitude differences. See also Extended Data Figure 4-2.

10.1523/ENEURO.0396-18.2019.f2-4 Figure 2-4 Download Figure 2-4, PDF file.

Concentration tracking (*CT*) responses differ on the first sniff of the static stimuli, but do not differ between the third sniff of step and static stimuli. (Fig. 3*B1*,*C*). A cell-odor pair is categorized as *CI* if the response on the first sniff of the static stimuli does not significantly differ between high and low, and the response on the third sniff of the concentration step stimulus does not differ from the third sniff of the two static stimuli. (Fig. 3*B2*,*C*). *ΔC_t_* sensitive responses differ on the third sniff of the *ΔC_t_* stimulus from the third sniff of both static stimuli. If after a positive change in concentration, the cell responded differently from its response to static high concentration, this cell-odor pair was categorized as *+ΔC_t_* (Fig. 3*B3*,*C*). *–ΔC_t_* cell-odor pairs gave a different response to the low concentration depending on the concentration in the preceding sniff (Fig. 3*B4*,*C*). In summary, 51% (*n* = 123) of cell-odor pairs responded to the odorants we presented. Of these responsive cell-odor pairs, 41% were *ΔC_t_*, 20% were *CT* and 39% were *CI* (Fig. 3*D*).

What is the cellular basis of *ΔC_t_* sensitivity? Are there dedicated “*ΔC_t_* cells” that represent concentration changes for all their effective odor stimuli, or does *ΔC_t_* sensitivity depend on odor identity? To approach this question, we compared the responses of each cell to different odors (Fig. 3*E*). An individual cell could belong to different response types for different odors. Importantly, cells with *ΔC_t_* sensitivity to one odor are not always *ΔC_t_* sensitive to other odors at the tested concentrations (Fig. 3*E*). Therefore, *ΔC_t_* sensitivity cannot be invariant to both odor identity and concentration. Further studies using a wider range of absolute concentrations will be necessary to determine whether there is invariance to either of these features.

### Contrast between concentrations depends on the stimulation history

In *ΔC_t_* responses (Fig. 2), the response to a given concentration depends on the concentration presented in the previous sniff. On the sniff after a concentration change, the difference between responses to different concentrations will be enlarged, thus enhancing the contrast for that sniff. Responses of M/T cells may encode odor stimuli either by changes in spike count or by changes in temporal profile without changes in spike count (Cury and Uchida, 2010; Shusterman et al., 2011). Our method of classifying responses is sensitive not only to changes in the total number of spikes within a sniff cycle but also to temporal redistribution of spikes within the cycle. To separately quantify which features of neuronal responses contribute to contrast enhancement, we compared the difference between responses to high and low concentrations when preceded by a step to the difference when preceded by the same concentration (Fig. 4*A*). We plotted full sniff spike count differences between the 3rd sniffs of the two static stimuli (|high-low|) against spike count differences between a dynamic step stimulus and the corresponding static stimulus (i.e., |dynamic-static|). In this visualization, the farther a cell-odor pair is from the diagonal, the stronger its contrast enhancement (Fig. 4*B*). Both *+ΔC_t_* and *–ΔC_t_* response populations showed contrast enhancement, with responses significantly shifted from the diagonal (Wilcoxon signed-rank test, *p* = 7.57*10^−5^ for *+ΔC_t_* and *p* = 8.64*10^−5^ for *–ΔC*_t_ responses), while the distributions for *CT* (Wilcoxon signed-rank test, *p* = 0.20, *n* = 25) and *CI* (Wilcoxon signed-rank test, *p* = 0.18, *n* = 49) responses are symmetric about the diagonal (Fig. 4*C*; Extended Data Fig. 4-1*A*).

10.1523/ENEURO.0396-18.2019.f4-1 Figure 4-1 Download Figure 4-1, PDF file.

10.1523/ENEURO.0396-18.2019.f4-2 Figure 4-2 Download Figure 4-2, PDF file.

**Figure 5.**
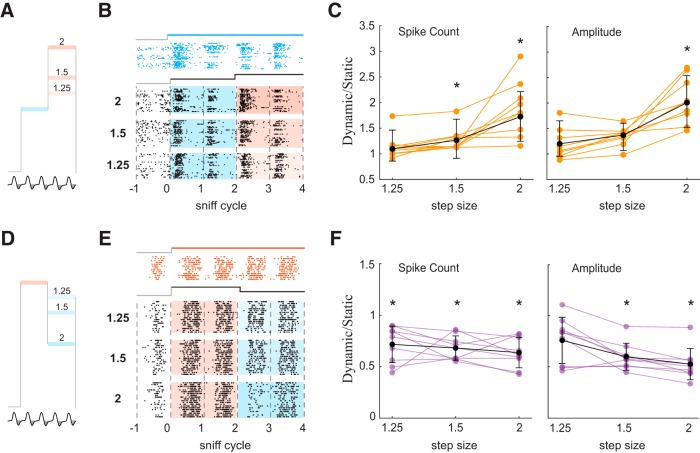
Contrast enhancement is proportional to the magnitude of concentration change step. ***A***, Stimulation with positive steps of different size. ***B***, Raster plots of M/T cell’s activity during L static and three LH dynamic step stimuli. ***C***, Normalized changes in spike count and amplitude of the response as function of step size. Orange lines are normalized changes for specific cell-odor pairs, black line is the mean ± SD change across all responsive cell-odor pairs. Asterisks mark statistically significant deviations from 1. ***D–F***, Same for negative steps.

10.1523/ENEURO.0396-18.2019.f5-1 Figure 5-1 Download Figure 5-1, PDF file.

To quantify how ΔC_t_ sensitivity enhances sub-sniff temporal differences between odor responses, we next performed the same comparison for differences in peak amplitude (peak firing rate; Fig. 4*D*; Extended Data Fig. 4-1*B*), a feature that reflects fast temporal patterning (Cury and Uchida, 2010; Shusterman et al., 2011). Peak amplitude difference distributions for *ΔC_t_* responses were significantly shifted from the diagonal (Wilcoxon signed-rank test, *p* = 2.41*10^−6^ for *+ΔC_t_* and *p* = 2.08*10^−8^ for *–ΔC_t_* responses), while for *CT* and *CI* responses the distributions were symmetric about the diagonal (Wilcoxon signed-rank test, *p* = 0.97 and *p* = 0.21, respectively). Thus, *ΔC_t_* sensitivity also increased contrast at the faster sub-sniff timescale. Lastly, to determine the trial by trial reliability of contrast enhancement by *ΔC_t_* responses, we used receiver operator characteristic (ROC) analysis (see Materials and Methods). In this analysis, *ΔC_t_* responses discriminated better between dynamic and static stimuli than between two static stimuli (Extended Data Fig. 4-2). These analyses demonstrate that *ΔC_t_* sensitivity enhances the contrast between concentrations, potentially facilitating detection of concentration changes.

### *ΔC_t_* sensitivity is step magnitude dependent

We next tested how *ΔC_t_* sensitivity depends on the magnitude of the concentration step. Because 2-fold concentration changes are in the range observed in turbulent plumes (Mylne and Mason, 1991; Crimaldi et al., 2002), and because firing rates fell to near zero in some *–ΔC_t_* responses (Fig. 2*C*; Extended Data Fig. 2-2), we tested responses to smaller concentration steps. In addition to the twofold steps used in the experiments above, we included a 1.5-fold and a 1.25-fold step, both LH and HL (Fig. 5*A*,*D*). To quantify *ΔC_t_* sensitivity, we took the ratio of the response to the dynamic stimulus to that of the static stimulus, for full sniff spike count as well as peak amplitude of the PSTH. *+ΔC_t_* responses (Fig. 5*B*) were largest for the 2-fold concentration increase, as expressed by the ratio of the response to the 3rd sniff of the dynamic stimulus (LH_3_) to that of the corresponding static stimulus (H_3_), both for spike count and peak amplitude (Fig. 5*C*). Across the population of *+ΔC_t_* responses, the two larger steps gave significant increases in spike count (*t* test; 1.25-fold change: *p* = 0.72; 1.5-fold change: *p* < 0.01; 2-fold change: *p* < 0.01), whereas only the largest step evoked a significant increase in peak amplitude: count (*t* test; 1.25-fold change: *p* = 0.5; 1.5-fold change: *p* = 0.06; 2-fold change: *p* < 0.001). For *–ΔC_t_* responses (Fig. 5*E*), spike counts were significantly reduced for all step sizes tested (*t* test; 1.25-fold change: *p* < 0.01; 1.5-fold change: *p* < 0.001; 2-fold change: *p* < 0.01; Fig. 5*F*), while peak amplitudes were significantly reduced for the two larger steps (*t* test; 1.25-fold change: *p* = 0.019; 1.5-fold change: *p* < 0.001; 2-fold change: *p* < 0.001; Fig. 5*F*). Thus, larger concentration steps give rise to stronger contrast enhancement.

### *ΔC_t_* sensitivity is independent of step duration

**Figure 6.**
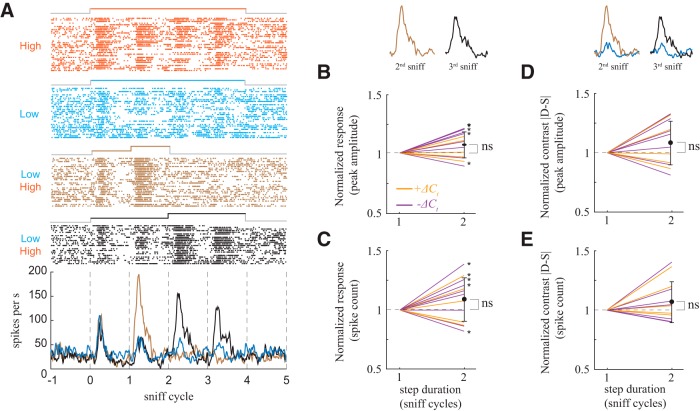
Contrast enhancement is independent of the duration of concentration change step. ***A***, Example of *+ΔC_t_* response to two stimuli of different step durations. Raster and PSTH plots of M/T cell response to static high concentration (orange), static low concentration (blue), low to high, step duration one sniff (brown), and low to high, two sniffs duration (black). PSTH of response for high static stimulus is not shown for clarity of visualization. ***B***, ***C***, Normalized changes in spike count and amplitude of the *+ΔC_t_* responses as function of step duration. Orange lines are normalized changes for *+ΔC_t_* responses, purple lines are for *–ΔC_t_* responses. Asterisks mark responses for which the one-sniff step response and the two-sniff step response differ significantly. ***D***, ***E***, Same as ***B***, ***C*** for changes in contrast (|dynamic-static|).

All responses we have shown thus far come from stimuli with steps lasting two sniffs. In natural environments, more rapid variations in odor concentration are common (Murlis et al., 1992). To test the extent to which *ΔC_t_* sensitivity is also evoked by briefer steps, we performed additional experiments in which concentration changed after one sniff (Fig. 6*A*). To quantify step duration dependent differences in *ΔC_t_* sensitivity, we normalized the peak amplitude (Fig. 6*B*) and spike count (Fig. 6*C*) of the *ΔC_t_* responses to the response for the one sniff step. While some responses were step duration dependent (5/13), across the population the differences were not significant (Wilcoxon test; Fig. 6*B*, *p* = 0.08; Fig 6*C*, *p* = 0.12). To characterize the extent to which contrast depends on step duration, as above we calculated the ratio of the dynamic response magnitude to static response magnitude for response amplitude (Fig. 6*D*) and spike count (Fig. 6*E*) and normalized this value to that of the one sniff long step. Across the population these differences were not significant (Wilcoxon test; *p* = 0.16; Fig. 6*E*, *p* = 0.41; Fig 6*D*).

### Concentration decoding depends on temporal pattern, while *ΔC_t_* decoding does not

**Figure 7.**
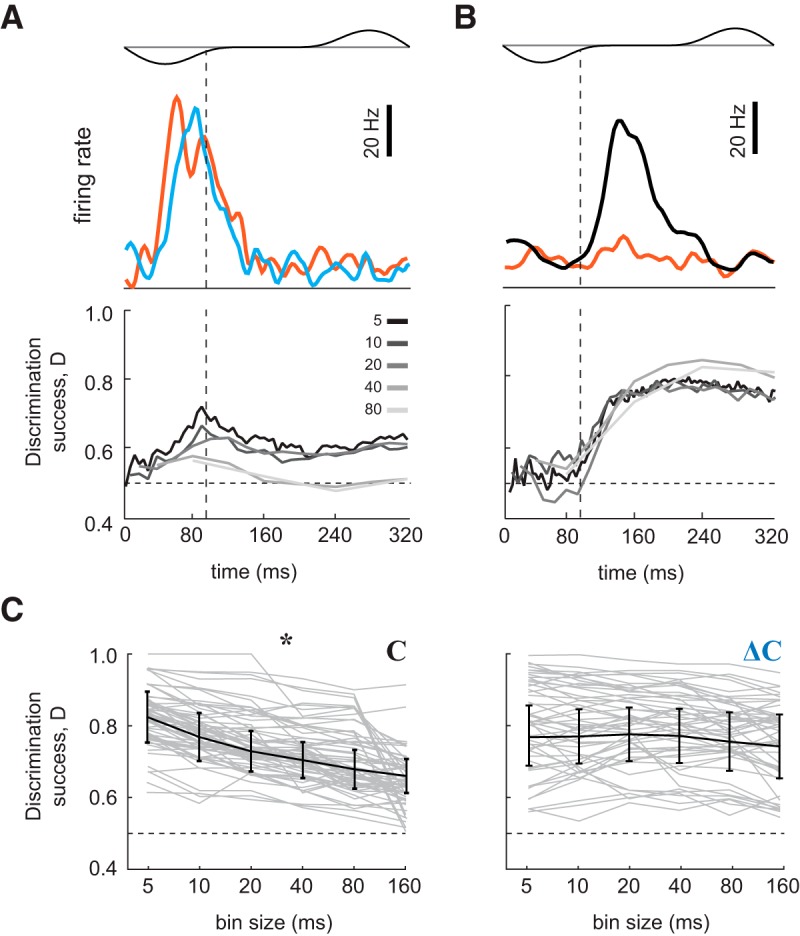
Discrimination among concentrations and changes in concentration by individual M/T cells. ***A***, top, PSTHs for a single neuron’s responses to two static stimuli (red: high concentration, blue: low concentration). Bottom, corresponding static stimuli discrimination success as a function of time. Vertical dashed lines indicate the end of the inhalation interval. Horizontal dashed lines indicate chance level performance. Different colored traces indicate discrimination success for different bin sizes. ***B***, top, PSTHs for a neuron’s responses to a high concentration static stimulus (red), and to a positive concentration step (black). Bottom, Corresponding static stimulus versus step stimulus discrimination success as a function of time. Different colored traces indicate discrimination success for different bin sizes. ***C***, Discrimination performance of a linear classifier between two odor concentrations (left) and between changes in concentration (right) over the 320-ms window, as a function of bin size. Gray lines are performances of individual neurons. Black line is mean ± SD. Asterisk (*) indicates significant change (one-way ANOVA; *p* < 0.01) in discrimination success as function of bin size.

In awake animals, M/T cell activity carries information about odor identity (Cury and Uchida, 2010; Shusterman et al., 2011) and intensity (Sirotin et al., 2015) at sub-sniff timescales. To compare how information about concentrations and about changes in concentration might be decoded by downstream olfactory areas, we performed discriminant analysis (see experimental procedures). We first evaluated the accuracy with which responses to two odor concentrations can be discriminated by cell-odor pairs with a *ΔC_t_* response (Fig. 7*A*). Classification of concentrations was performed on concatenated vectors of firing rates with multiple bin sizes: 5, 10, 20, 40, 80, and 160 ms. Concentration classification performance depended on bin size: smaller bin sizes yielded better discrimination (one-way ANOVA; *p* < 0.01; Fig. 7*C*). Thus, information about odor concentration can be read out most accurately from fine timescale temporal patterns. Using the same classification procedure, we next evaluated whether decoding of concentration changes by the same *ΔC_t_* cell-odor pairs similarly depends on temporal resolution (Fig. 7*B*). This analysis indicates that decoding of concentration changes is invariant across the full range of bin sizes (one-way ANOVA, *p* = 0.22; Fig. 7*C*). These findings suggest that downstream neurons decode concentration and *ΔC_t_* via different mechanisms.

## Discussion

Studies of freely moving animals have established the importance of odor concentration dynamics in guiding olfactory navigation (Khan et al., 2012; Catania, 2013; Jones and Urban, 2018). While these paradigms have revealed behavioral strategies, odor stimuli in an open field cannot currently be precisely controlled or measured. Without precise knowledge of the stimulus, neuronal responses are difficult to interpret. To achieve precise stimulus control, we have developed a novel system for presenting rapidly changing odor concentration stimuli to head-fixed mice.

Our concentration step stimuli have revealed three response types across cell-odor pairs: (1) *CT* responses, in which firing rate is proportional to odor concentration on the current sniff, irrespective of concentration in past sniffs; (2) *CI* responses, in which firing rate does not vary across the range of presented odor concentrations; and (3) concentration change sensitive (*ΔC_t_*) responses, in which firing rate depends not only on the currently-sniffed concentration, but also that of the previous sniff. A given M/T cell can give different response types to different odorants. Thus, it does not appear that these response types map onto particular cell types.

*ΔC_t_* responses enhance the contrast between different concentrations, both in fine and coarse timescales. This contrast enhancement scales with the concentration step magnitude but does not depend on the duration of the step. Lastly, we show that decoding of concentration steps does not depend on the duration of time bins: reading fine timescale features does not improve classification performance.

Taken together, we have obtained the first evidence that neurons in the mammalian olfactory system represent inter-sniff changes in odor concentration. Such temporal contrast enhancement is widespread in other sensory modalities, consistent with the paramount importance of sensing stimulus dynamics. Furthermore, we find that this representation is already present near the sensory periphery, in the olfactory bulb. Computing *ΔC_t_* near the periphery allows the signal to be broadcast to the OB’s numerous targets in the cortex.

### Neuronal mechanisms of *ΔC_t_* sensitivity

Invertebrate olfactory organs sample incoming odors continuously, so that their OSNs are directly exposed to gradients of odor concentration (Nagel and Wilson, 2011; Kim et al., 2015; Schulze et al., 2015), as well as intermittent intensity fluctuations found in plumes (Vickers et al., 2001). In contrast, terrestrial vertebrates such as mice sample odors intermittently. To compare the intensities of the previous and the current inhalation, the animal must preserve a representation of the previous concentration during the exhalation interval. A simple way in which information can persist over time is through history-dependent adaptation. Adaptation allows cells to match their limited dynamic range to the distribution of stimulus intensities in the environment (Kohn, 2007). We propose that the function of *ΔC_t_* responses is to shift the dynamic range of olfactory neurons to increase sensitivity to concentrations close to the recently inhaled stimulus. A similar adjustment of dynamic range has been observed for motion processing in the insect visual system (Fairhall et al., 2001). Mechanistically, shifts in dynamic range may be implemented via intrinsic neuronal properties, such as spike threshold adaptation (Henze and Buzsáki, 2001; Itskov et al., 2011) Alternatively, *ΔC_t_* sensitivity may be achieved by circuit mechanisms, such as intrabulbar interactions (Shepherd and Greer, 1998; Wachowiak and Shipley, 2006; Burton, 2017) or cortical feedback (Luskin and Price, 1983; Li and Hopfield, 1989; Boyd et al., 2012; Markopoulos et al., 2012; Boyd et al., 2015; Otazu et al., 2015).

A cell with *ΔC_t_* sensitivity to one odor can have a different response type to another effective odor. This eliminates the possibility that a dedicated population of “*ΔC_t_* cells” represents *ΔC_t_*irrespective of odor identity and absolute concentration. Similarly, malleable stimulus selectivity has been observed in other sensory systems. For example, in the retina, although it is widely accepted that retinal ganglion cells consist of dedicated cell types with selectivity for a particular visual feature, recent work challenges this view (Rivlin-Etzion et al., 2018; Wienbar and Schwartz, 2018). Identified ON or OFF retinal ganglion cells can change their polarity based on stimulation outside the receptive field (Geffen et al., 2007) and ambient light levels (Tikidji-Hamburyan et al., 2015). Direction-selective ganglion cells can reverse their preferred direction of motion depending on recent stimulus history (Rivlin-Etzion et al., 2012). Thus, even classic “feature detector” cell types of the retina can change their selectivity under different conditions. As with other sensory features, understanding *ΔC_t_*processing will require a more thorough exploration of stimulus space, in as close to natural conditions as possible.

### Potential relevance of *ΔC_t_*sensitivity in the natural environment

Odor concentration gradients are critical for odor source localization (Murlis et al., 1992). Mice must locate odor sources in various airflow conditions, which will largely determine the spatiotemporal statistics of odor concentration. Turbulence disrupts concentration gradients emanating from a distant odor source (Murlis et al., 1990, 1992; Weissburg, 2000). However, even in turbulent flow, gradients, and therefore *ΔC_t_*, become increasingly informative closer to the source (Justus et al., 2002; Riffell et al., 2008; Gire et al., 2016). Therefore, when following a plume from a nearby source (Catania, 2013; Gire et al., 2016), or when tracking a depositional odor trail (Khan et al., 2012; Jones and Urban, 2018), *ΔC_t_*signals can guide the nose.

In the real world, there may also be odor fluctuations faster than the inhalation time scale. We argue that temporal changes in odor concentration on the sub-sniff scale are not relevant, due to several slow processes. First, based on the physics of the nasal cavity, odor fluctuations will be low pass filtered (Doebelin, 1990). Second, the odorant molecules must transition from air to liquid and diffuse through the mucus (Hahn et al., 1994). Lastly, the flicker fusion frequency of mouse OSNs *in vitro* is 3–5 Hz (Ghatpande and Reisert, 2011). Because of these slow processes, we think it is unlikely that sub-sniff timescale changes in odor concentration are available to the olfactory system.

Vertebrates sense gradients by stereo (inter-naris) and serial (inter-sniff) comparisons (Rajan et al., 2006; Catania, 2013). Because the nares are close together, stereo comparison should be most informative near an odor source, where odor gradients are steep. Shallower gradients, farther from a source, may require the inter-sniff comparison, since the distance between sampling locations can be larger than the inter-naris distance (Catania, 2013). In a turbulent environment with noisy gradients (Gire et al., 2016), comparison over more than two sniff cycles may be required. While stereo comparisons have been studied both behaviorally (Rajan et al., 2006; Porter et al., 2007; Catania, 2013) and electrophysiologically (Rajan et al., 2006; Kikuta et al., 2010), the serial component, which should dominate over a wider range of distances, has not been explored. Our study demonstrates a neural representation of *ΔC_t_*. We propose that this representation contributes to olfactory search in natural olfactory scenes.

## Synthesis

Reviewing Editor: Anna Menini, SISSA (International School of Advances Studies) Decisions are customarily a result of the Reviewing Editor and the peer reviewers coming together and discussing their recommendations until a consensus is reached. When revisions are invited, a fact-based synthesis statement explaining their decision and outlining what is needed to prepare a revision will be listed below. The following reviewer(s) agreed to reveal their identity: Justus Verhagen.

Both reviewers highlight the importance of this work that systematically shows for the first time that the olfactory bulb encodes inter-sniff changes in odor concentration. To obtain high-quality data, precisely controlled odor stimuli were delivered to awake, head-fixed mice using a novel stimulation system constructed to rapidly change odor concentrations between inhalations. This excellent manuscript will be further improved by addressing the specific comments listed below.

Reviewer #1

In this manuscript the authors demonstrate for the first time systematically that the bulb encodes concentration changes on a sniff by sniff basis in the awake animal. They show that mitral/tufted cells in the OB can respond to concentration change in a different way than to absolute concentration. They carefully control stimuli, have built an exceptionally well-controlled olfactometer and obtain high quality data in the awake animal and carefully and comprehensively analyze their results. In general this is done very clearly, with good examples and summary data, and is very clearly written.

This is a very interesting and important phenomenon for everyone in the field given the potential behavioral relevance for e.g. odor navigation theory. One could argue that the current manuscript remains descriptive at this point but in my opinion it is an important starting point for investigating concentration change encoding and the authors provide a good discussion of potential mechanisms.

My only somewhat major comment is that I am confused with the very precise definition of the different categories - this needs improved presentation in text and figures:

(1) I am a bit confused with the precise definition by whether the separation into DeltaCT, CI and CT indeed covers the entire set of possibilities with three comparisons there are 8 possibilities, CT covers 2 (difference between dynamic and low can either be sig or ns), CI one, Delta CT covers two again (comparison of initial response can be either ns or sig) - this amounts to 5, i.e. three combinations missing -did the authors observe any cell-odor pair where for example on the third cycle the response to the step was significantly different from the low stimulus but not from the high stimulus but the initial (first sniff) response to high and low was not significantly different? To my understanding this would not fall into any of the categories illustrated in 3B? Maybe the authors can clarify. Also, the examples in Fig 3B are very illustrative, providing one for minus Delta CT in the same format would be helpful.

(2) Can the authors comment on the change in firing rate for increased and decreased concentrations (for the Delta CT cells)? All examples shown suggest that firing rate consistently increased with positive concentration changes (for the delta CT cells). Is that true? I might be confuse with the definitions here - Extended data fig 2-2 shows an alleged -DeltaCT cell. However, the response to the Low-High stimulus (purple) clearly looks significantly different on the third sniff compared to the High and Low response. Isn't that the definition of a plus Delta CT (line 276: “If after a positive change in concentration, the cell responded differently from its response to static high concentration, this cell-odor pair was categorized as +delta CT”) ? Or should the more precise definition of a plus Delta CT cell-odor pair be that the response to a LH step is significantly LARGER than the response to static Low/static high??

Minor comments / queries for clarification:

(3) DO the authors have data or can they speculate about larger concentration differences? As the Delta-C used is relatively small (twofold) in my opinion the term “concentration invariant” is a bit of a stretch. This should be discussed.

(4) For helping the reader to assess their own understanding it would help if the examples shown are marked out in the summary plots, e.g. the examples from Fig 3B in Fig 4C/D

(5) Fig 3D: This only summarizes how ΔC responses diversify on different odors, why not show for other types of response?

Reviewer #2

General:

This ms reports for the first time MTC responses to in- and decreases in odor concentration between subsequent sniffs in awake head-fixed mice, among responses that merely track odor concentration independently of history and concentration invariant responses. The approach is novel, allowing rapid changes in odor concentration between inhalations. The finding is important as it provides an obvious means for the brain to encode odor plume dynamics which may inform about odor source location. To this reviewer the data are compelling and very significant.

Major:

1.

The finding that there are no generalized dCt cells in the recorded population is both interesting and surprising to me. A cell may show dCt to 1 odor but only to 20% of others (Fig 3D). If so, how are changes in concentration in naturalist odors, i.e. mixtures, encoded? (Also L417-429)

Also, what possible mechanism allows for this odor-dependent dCt sensitivity? (I note the discussion is quite thorough generally but I missed this interesting issue), e.g. L400-412.

It'd further be interesting to see the distribution of dCt responses for the cells that were tested with 3 odors (the majority, 92 cells w 242 cell-odor pairs), and inversely for odors tested on more than 10 cells. This to o augment Fig 3D which only shows this in general for some 2nd response and get better idea what cells do.

2.

Do differences in sniffing explain any of the different response types? E.g. is sniff amplitude (Pauc) enhanced during dCt responses?

Minor:

L110: why can odor not enter the nose during exhalation? The lungs etc. only partly scavenge inhaled odors.

L156-160 & Efig 4-2 A3, B3, C3: how was ROC created? What's on x and y axes, what does each point mean? How's is false positive rate calculated? In contrast, this is clear for Fig 4.

L195: Only 2 mice for behavioral experiments..not sure why recordings weren't all done during go-no-go task? Not critical but perhaps state. Also Efig1-2 ~65% correct during no odor is weird, perhaps explaining more than current (which doesn't explain it imo).

L228: turbulent plumes show very large dynamics (intermittency), not sure where the ‘twice’ comes from? Nonetheless it shows that even this subtly change can be very effective.

Fig 2: I count n=26 in E and n=17 in F, should be 28 and 21? Missing data? Also, general issue, do the -dCt responses suppress below air only sniff response?

Fig 3: how reliable/repeatable is a particular dCt response? Were repeated trials made? ANOVA? How does L316-321 address this?

Fig 5: the steps shown in A and D do not seem to be 2, 1.5 and 1.25...?

Fig7AB: is each n=1 example neuron?

Text edits:

L2 dynamic

L6 represents

L26 ._Vertebrates

Stats table: p=0.0.8 and “- p=0.08”

L143 “after THE step to the static odor response” ..not sure that's what you want to say

L299-300 the difference V.S. when?

Fig 4D amplitude is peak firing rate? Also as elsewhere colors orange and blue (yellow and purple?)

L442 Doebelin not in Refs

L554 ref?

L665 green, orange (note yellow and purple; match A-D?)
