# Supplementary material for: Odor Concentration Change Coding in the Olfactory Bulb
Source: eNeuro. 2019 Feb 27;6(1):ENEURO.0396-18.2019. doi: 10.1523/ENEURO.0396-18.2019 (PMC6397952; doi:10.1523/ENEURO.0396-18.2019)
Supplement: Figure 1-1 — Download Figure 1-1, PDF file. [file sup_enu-eN-NWR-0396-18-s02.pdf]

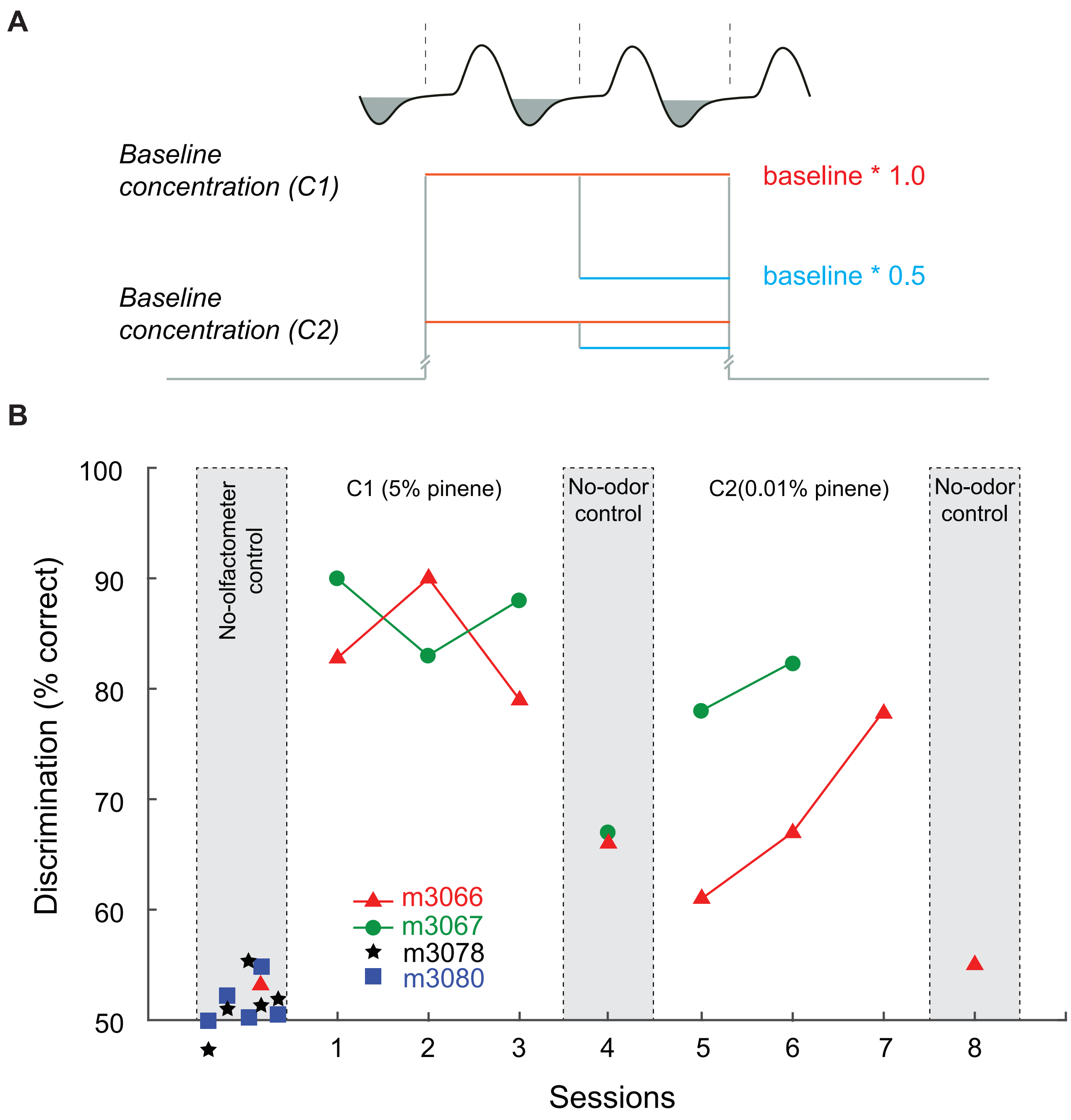

Extended data Figure 1-1. Concentration change behavioral paradigm. A. Design of the behavioral paradigm. Stimulation events are triggered in real time from the sniff signal. Exhalation onsets are detected by positive-going zero-crossings in the sniff signal. The first exhalation triggers odor onset to a baseline concentration, the second exhalation triggers the concentration step of different sizes, and the third exhalation triggers the odor offset. B. Behavioral performance. After initial training (not shown), mice were run in control sessions where the air input bypasses the olfactometer. Chance performance in these sessions eliminates the possibility that non-olfactory cues are driving behavioral performance. Mice report two-fold concentration steps of pinene with high performance at a high odor concentration (1:20 mineral oil dilution; 1:10 air dilution). Following these sessions, the mice were run in control sessions where air input was run through a blank vial in the olfactometer. Mice perform above chance, most likely due to odor lingering in the olfactometer. At a lower concentration (1:10000 liquid dilution; 1:10 air dilution), mice can still report concentration steps at a high level of performance.
