# Supplementary material for: Odor Concentration Change Coding in the Olfactory Bulb
Source: eNeuro. 2019 Feb 27;6(1):ENEURO.0396-18.2019. doi: 10.1523/ENEURO.0396-18.2019 (PMC6397952; doi:10.1523/ENEURO.0396-18.2019)
Supplement: Figure 1-2 — Download Figure 1-2, PDF file. [file sup_enu-eN-NWR-0396-18-s10.pdf]

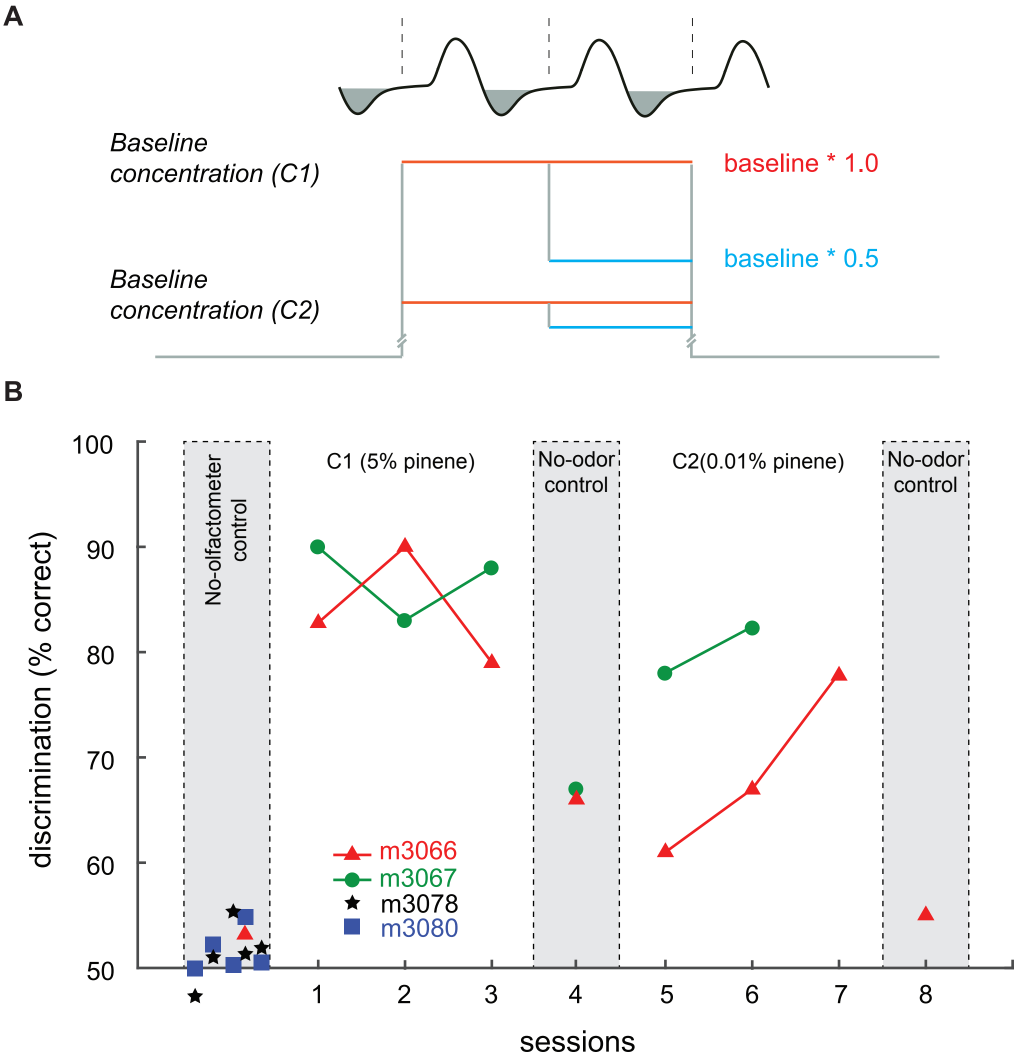

Extended data Figure 1-2. Concentration change behavioral paradigm.

A. Design of the behavioral paradigm. Stimulation events are triggered in real time from the sniff signal. Exhalation onsets are detected by positive-going zero-crossings in the sniff signal. The first exhalation triggers odor onset to a baseline concentration, the second exhalation triggers the concentration step of different sizes, and the third exhalation triggers the odor offset. **B.** Behavioral performance. After initial training (not shown), mice were run in control sessions where the air input bypasses the olfactometer. Chance performance in these sessions eliminates the possibility that non-olfactory cues are driving behavioral performance. Mice report two-fold concentration steps of pinene with high performance at a high odor concentration (1:20 mineral oil dilution; 1:10 air dilution). Following these sessions, the mice were run in control sessions where air input was run through a blank vial in the olfactometer. Mice perform above chance, most likely due to odor lingering in the olfactometer. At a lower concentration (1:10000 liquid dilution; 1:10 air dilution), mice can still report concentration steps at a high level of performance.
