# Supplementary material for: Odor Concentration Change Coding in the Olfactory Bulb
Source: eNeuro. 2019 Feb 27;6(1):ENEURO.0396-18.2019. doi: 10.1523/ENEURO.0396-18.2019 (PMC6397952; doi:10.1523/ENEURO.0396-18.2019)
Supplement: Figure 2-1 — Download Figure 2-1, PDF file. [file sup_enu-eN-NWR-0396-18-s06.pdf]

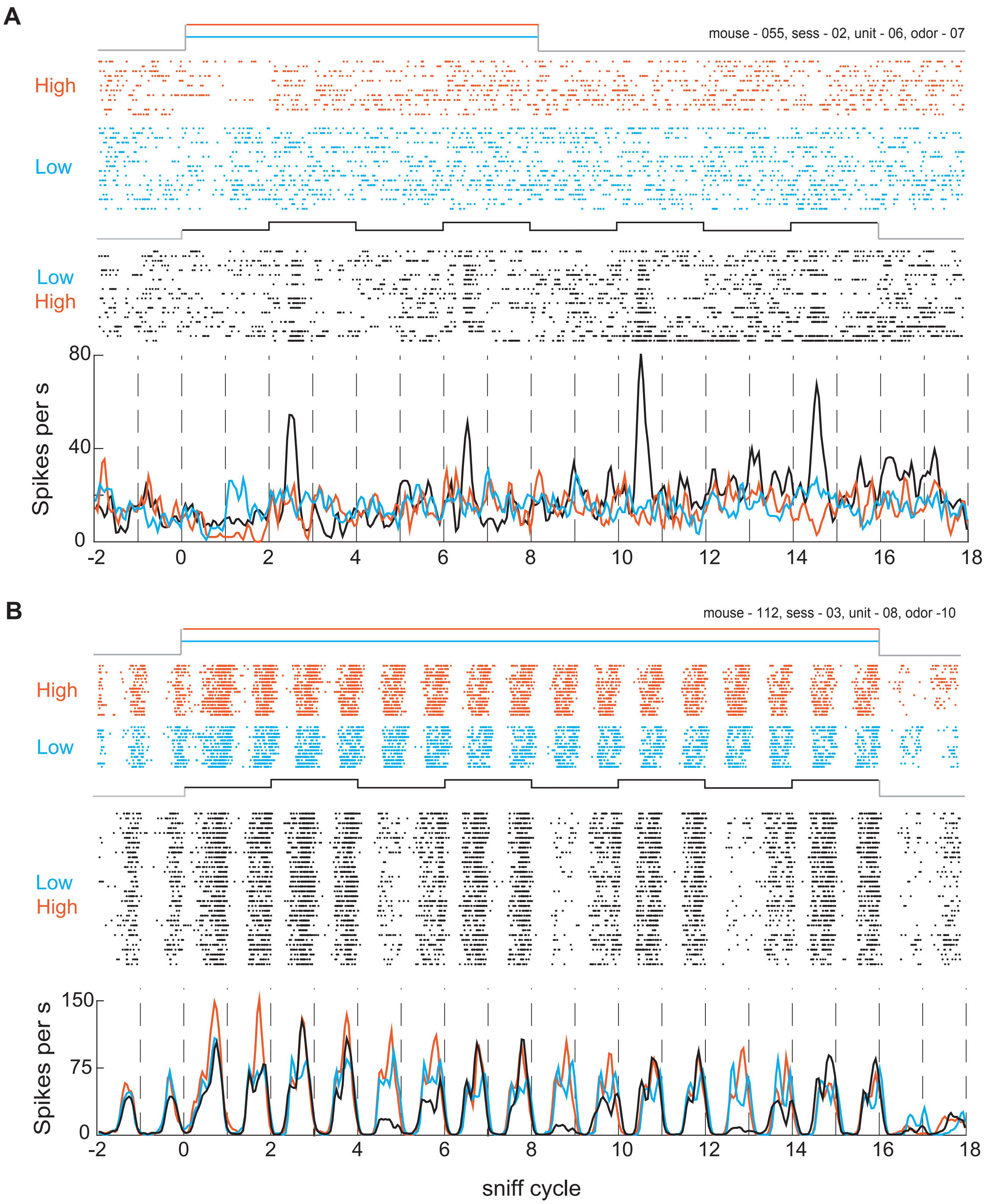

Extended data Figure 2-1. ΔC_t-sensitive responses to prolonged concentration step stimuli.

A. Raster and PSTH plots of M/T cell response to static high concentration (orange), static low concentration (blue) and prolonged concentration step (black) stimuli. This cell-odor pair gives an excitatory response to both static stimuli, but greatly reduces its response after an upward concentration step. **B.** Same as (A), but for a different cell odor pair. This cell-odor pair gives little response to static stimuli, and responds strongly for decreasing concentration steps.
