# Supplementary material for: Odor Concentration Change Coding in the Olfactory Bulb
Source: eNeuro. 2019 Feb 27;6(1):ENEURO.0396-18.2019. doi: 10.1523/ENEURO.0396-18.2019 (PMC6397952; doi:10.1523/ENEURO.0396-18.2019)
Supplement: Figure 2-2 — Download Figure 2-2, PDF file. [file sup_enu-eN-NWR-0396-18-s07.pdf]

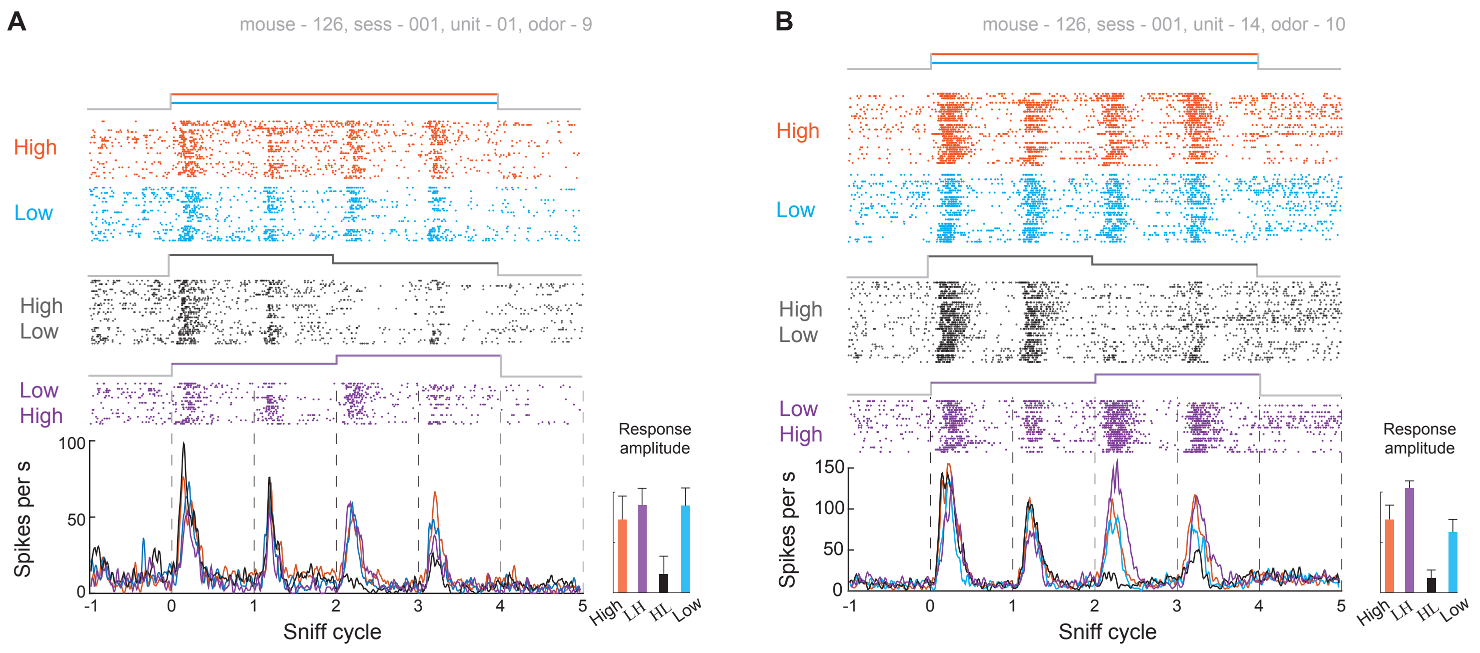

Extended data Figure 2-2. M/T cells responsive to changes in odor concentration.
A. Example of -ΔC_t response. Raster and PSTH plots of M/T cell response to static high concentration (orange), static low concentration (blue), low to high (purple) and high to low (black). Bar graph on right shows peak response amplitudes on the third sniff cycle for each stimulus. Error bars indicate standard deviation. **B.** Example of cell-odor pair responsive to both +ΔC_t and -ΔC_t stimulation.
