# Supplementary material for: Odor Concentration Change Coding in the Olfactory Bulb
Source: eNeuro. 2019 Feb 27;6(1):ENEURO.0396-18.2019. doi: 10.1523/ENEURO.0396-18.2019 (PMC6397952; doi:10.1523/ENEURO.0396-18.2019)
Supplement: Figure 2-3 — Download Figure 2-3, PDF file. [file sup_enu-eN-NWR-0396-18-s08.pdf]

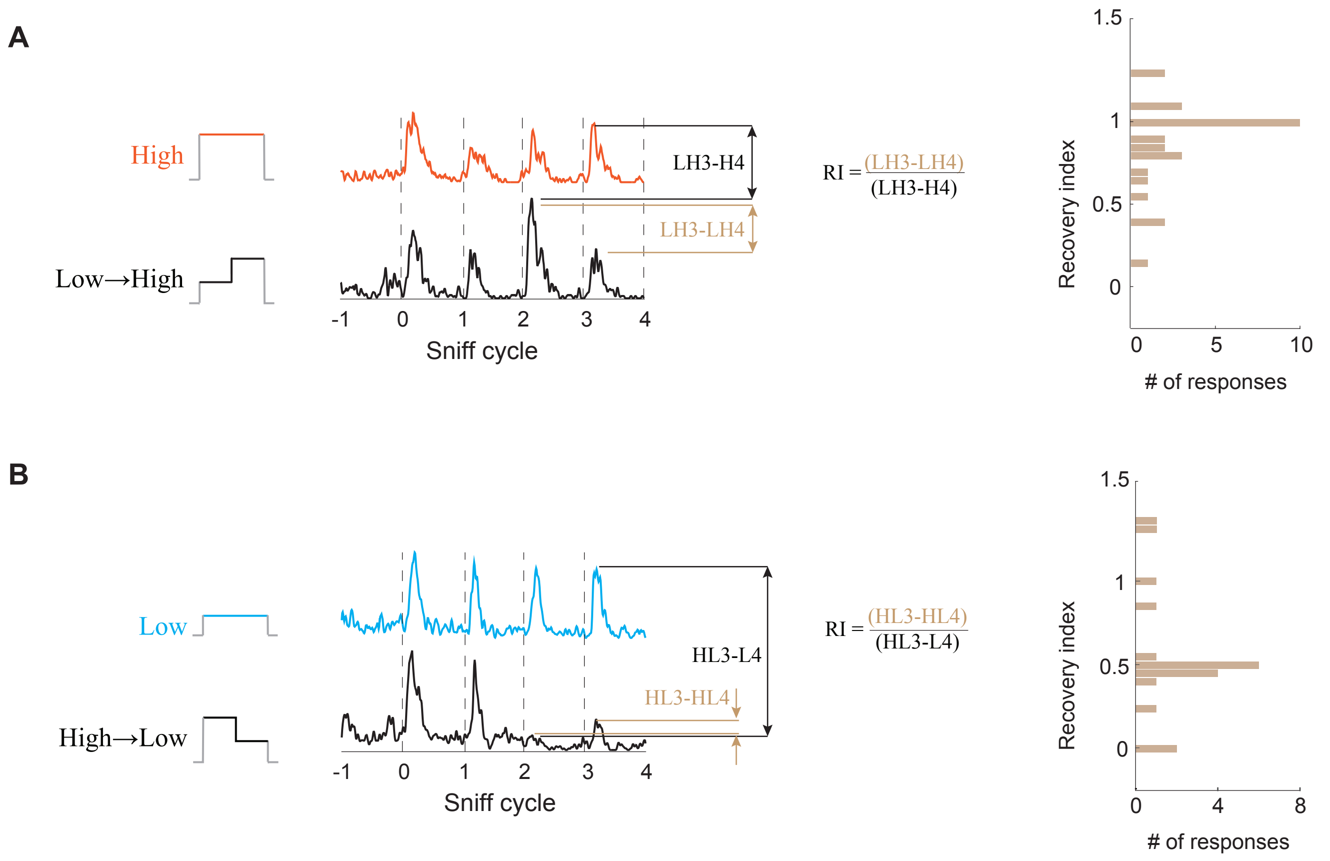

Extended data Figure 2-3. Response recovery index.
A. *Left*. Schematic of recovery index for $+\Delta C_t$ responses. It is the ratio between change of response across two consecutive sniff cycles after the concentration step (LH3-LH4) to the difference between the ΔC_t response and the response on the matching static stimulus (LH3-H3). *Right*. Distribution of recovery indices (same as Fig. 2E)
B. *Left*. Schematic of recovery index for $-\Delta C_t$ responses: (HL3-HL4) / (HL3-L3). *Right*. Distribution of recovery indices (same as Fig. 2F).
