# Supplementary material for: Odor Concentration Change Coding in the Olfactory Bulb
Source: eNeuro. 2019 Feb 27;6(1):ENEURO.0396-18.2019. doi: 10.1523/ENEURO.0396-18.2019 (PMC6397952; doi:10.1523/ENEURO.0396-18.2019)
Supplement: Figure 2-4 — Download Figure 2-4, PDF file. [file sup_enu-eN-NWR-0396-18-s03.pdf]

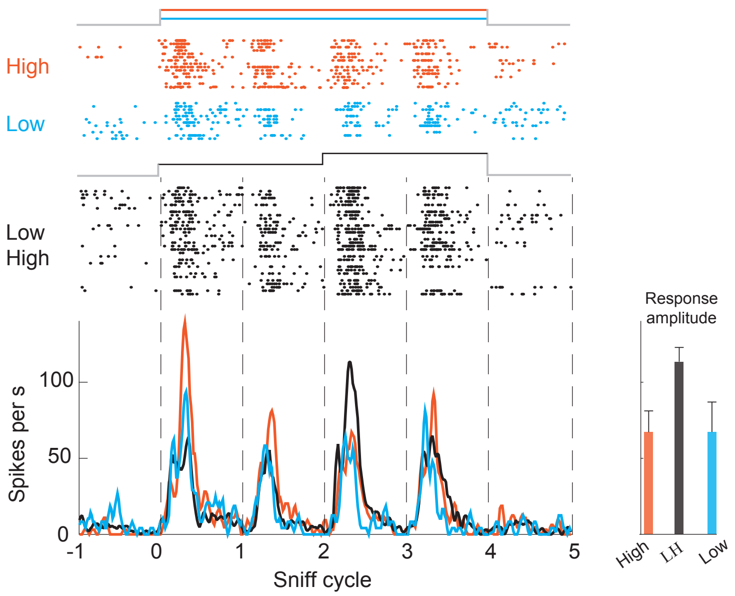

Extended data Figure 2-4. An example +ΔC_t response with strong adaptation.

Raster and PSTH plots of M/T cell response to static high concentration (orange), static low concentration (blue), and low to high (black). Bar graph on right shows peak response amplitudes on the third sniff cycle for each stimulus. Error bars indicate standard deviation. This example illustrates the importance of comparing the same sniff cycle after stimulus onset. The response to H adapts strongly, such that the 3rd sniff cycle response is significantly lower than the 1st sniff response. Even though the 3rd sniff response to LH is also lower than the 1st sniff response to H, it is significantly larger than the 3rd sniff response to H. Thus, it still contains information about change and by our definition is a ΔC_t response.
