# Supplementary material for: Odor Concentration Change Coding in the Olfactory Bulb
Source: eNeuro. 2019 Feb 27;6(1):ENEURO.0396-18.2019. doi: 10.1523/ENEURO.0396-18.2019 (PMC6397952; doi:10.1523/ENEURO.0396-18.2019)
Supplement: Figure 4-1 — Download Figure 4-1, PDF file. [file sup_enu-eN-NWR-0396-18-s04.pdf]

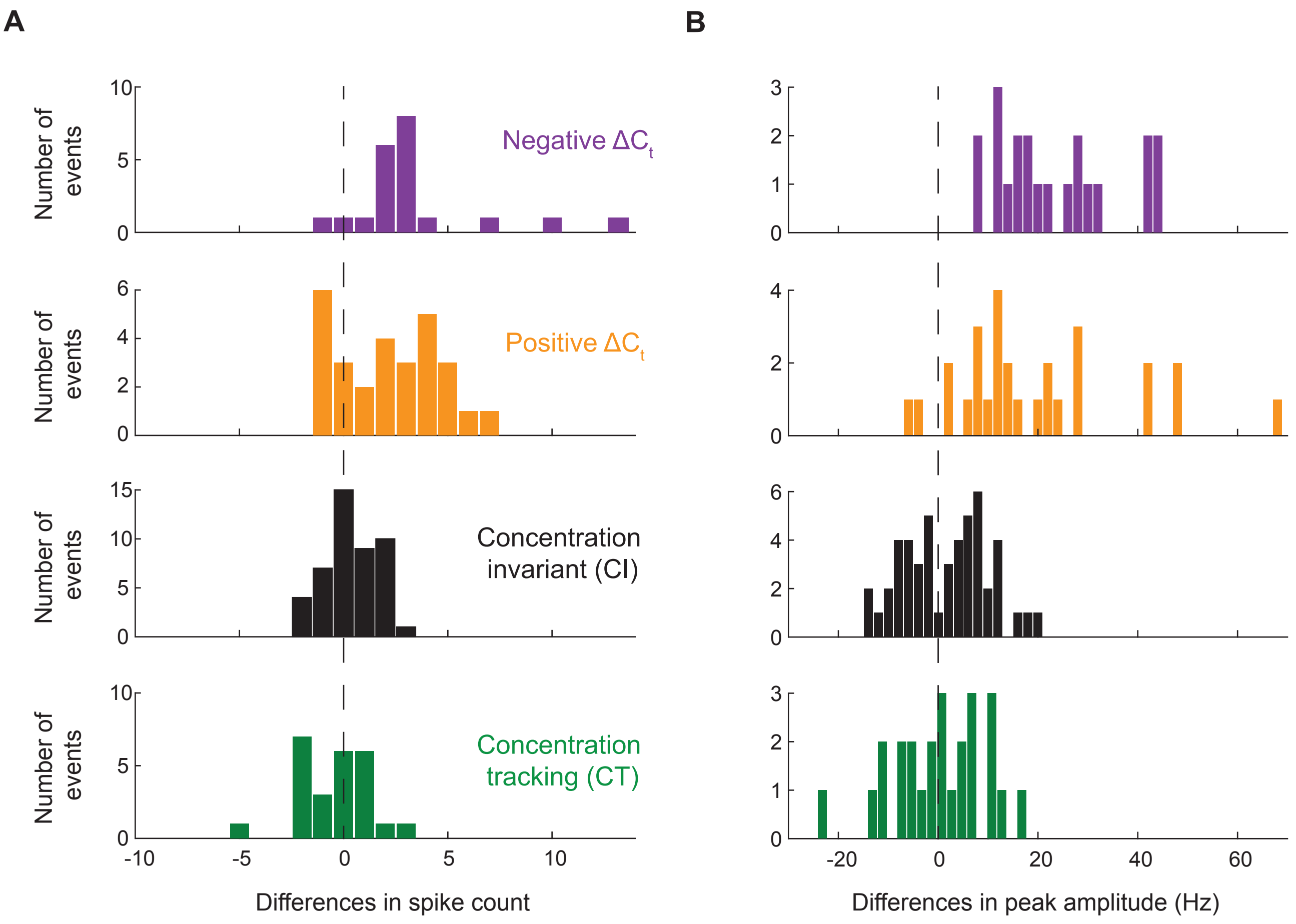

Extended Data Figure 4-1. Distributions of differences in amplitude and spike count due to contrast increase.

A. Distributions of the spike count differences between two static stimuli against differences between dynamic and static stimuli, on the 3rd sniff cycle. $-\Delta C_t$, $+\Delta C_t$, *CI*, and *CT*, marked by purple, orange, black, and green color, respectively. This subplot complements Fig. 4C. **B.** Same as (A) for peak amplitude differences. This complements Fig. 4D.
