# Supplementary material for: Odor Concentration Change Coding in the Olfactory Bulb
Source: eNeuro. 2019 Feb 27;6(1):ENEURO.0396-18.2019. doi: 10.1523/ENEURO.0396-18.2019 (PMC6397952; doi:10.1523/ENEURO.0396-18.2019)
Supplement: Figure 4-2 — Download Figure 4-2, PDF file. [file sup_enu-eN-NWR-0396-18-s05.pdf]

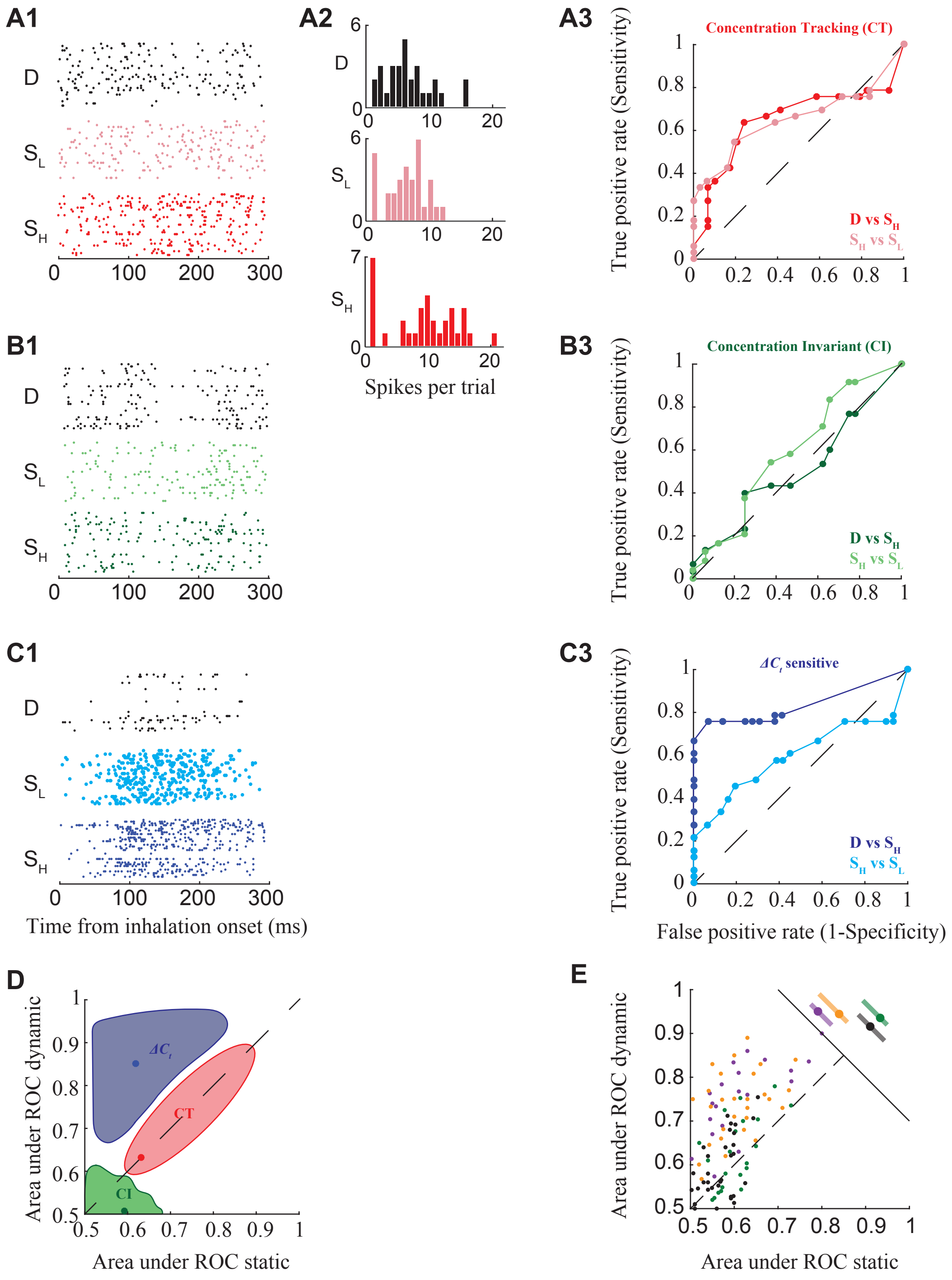

Extended Data Figure 4-2. ROC analysis separates different response types.

A1. Raster plots of a concentration tracking (*CT*) cell-odor pair on the 3rd sniff cycle to static high (red) and static low (pink) and step (black) stimuli. **A2.** Full sniff spike count histogram for each response from **A1**. **A3.** ROC curves between the static odor stimuli: high and low (pink), and between step and corresponding static (red). **B.** Same as **A**, but for an example concentration invariant (*CI*) response. **C.** Same as **A**, but for an example ΔC_t sensitive response. **D.** Expected distribution of responses. *CT* responses will be distributed within the green area, *CT* responses will be distributed along diagonal, and ΔC_t responses will be distributed above diagonal. **e.** Scatter plot of ΔC_t discriminability against static stimulus discriminability. *CI*, *CT*, $+\Delta C_t$ and $-\Delta C_t$ marked by black, green orange and blue color, respectively. Adjacent panel shows the means and STDs of the response discriminability differences.
