# Supplementary material for: Odor Concentration Change Coding in the Olfactory Bulb
Source: eNeuro. 2019 Feb 27;6(1):ENEURO.0396-18.2019. doi: 10.1523/ENEURO.0396-18.2019 (PMC6397952; doi:10.1523/ENEURO.0396-18.2019)
Supplement: Figure 5-1 — Download Figure 5-1, PDF file. [file sup_enu-eN-NWR-0396-18-s01.pdf]

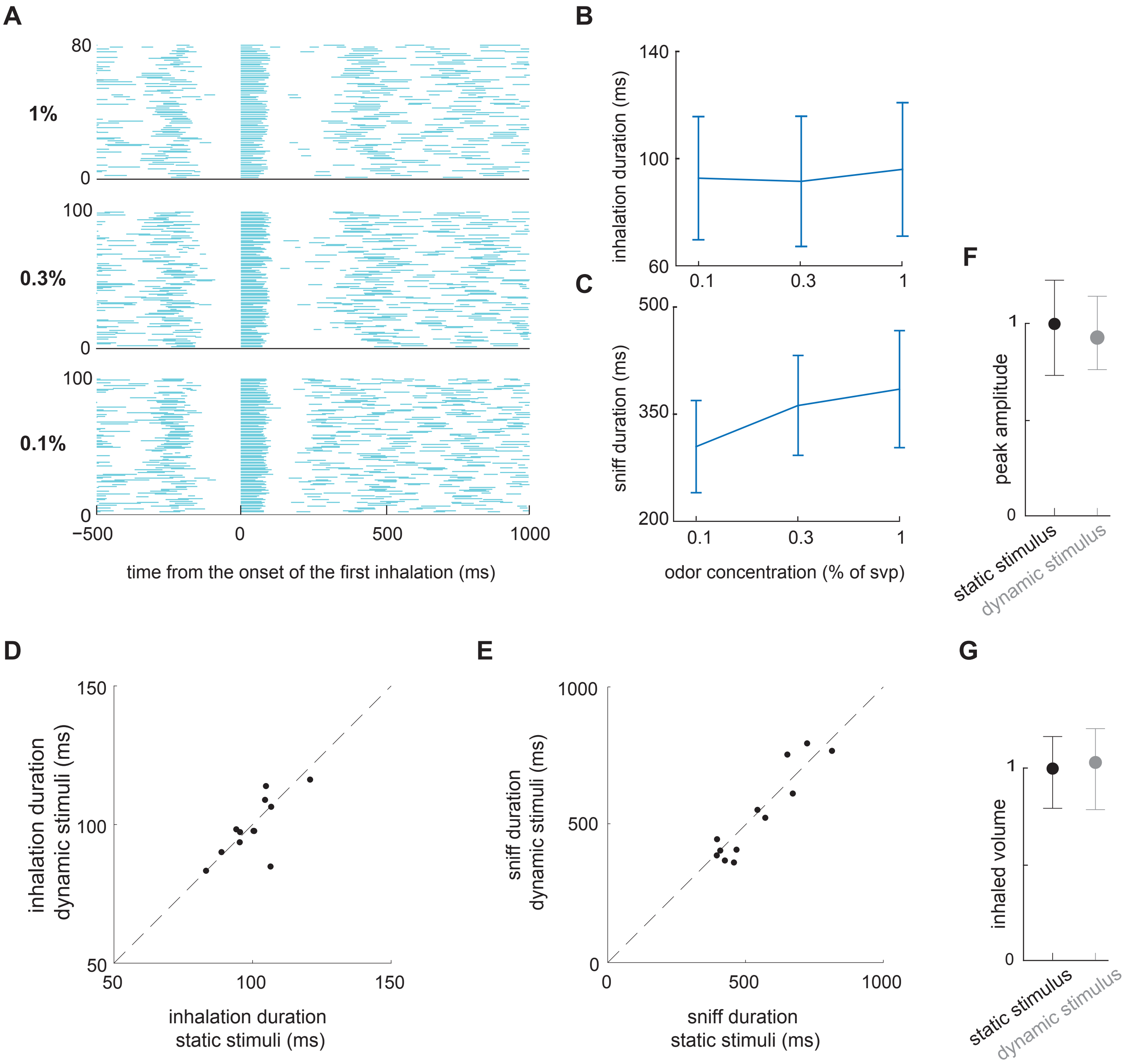

Extended data Figure 5. Variation in sniff parameters with static and dynamic stimuli.
A. Raster plots of inhalations to 3 concentrations of Amyl Acetate, aligned by the onset of the first odorized inhalation. Blue lines represent the inhalation intervals, while white space indicates exhalation intervals. **B.** Mean and standard deviation of inhalation duration distributions on 1st odorized cycle as function of odor concentration. **C.** Same as (B) for whole sniff durations. **D.** Comparison of inhalation duration on the 3rd sniff cycle for static vs. dynamic stimuli of Amyl Acetate. Each dot represents different session. **E.** Same as (D) for whole sniff duration. **F.** Comparison of peak amplitude on the 3rd sniff cycle for static vs. dynamic stimuli. Peak amplitude of both dynamic and static sniffs during each session were normalized to an average peak ampliture of static sniff cycle. **G.** Same as (F) for inhaled volume.
